# Optimisation of Ethanol-Reflux Extraction of Saponins from Steamed *Panax notoginseng* by Response Surface Methodology and Evaluation of Hematopoiesis Effect

**DOI:** 10.3390/molecules23051206

**Published:** 2018-05-17

**Authors:** Yupiao Hu, Xiuming Cui, Zejun Zhang, Lijuan Chen, Yiming Zhang, Chengxiao Wang, Xiaoyan Yang, Yuan Qu, Yin Xiong

**Affiliations:** 1 Faculty of Life Science and Technology, Kunming University of Science and Technology, Kunming 650500, China; hypflygo@163.com (Y.H.); cuisanqi37@163.com (X.C.); 18380802826@163.com (Z.Z.); chen13990207@163.com (L.C); jr93586@163.com (Y.Z.); wcx1192002@126.com (C.W.); yangxiaoyan9999@163.com (X.Y.); quyuan2001@126.com (Y.Q.); 2 Yunnan Key Laboratory of *Panax notoginseng*, Kunming University of Science and Technology, Kunming 650500, China; 3 Laboratory of Sustainable Utilization of *Panax notoginseng* Resources, State Administration of Traditional Chinese Medicine, Kunming University of Science and Technology, Kunming 650500, China

**Keywords:** steamed *Panax notoginseng*, saponins, extraction, optimization, antioxidant activity, response surface methodology, hematopoiesis

## Abstract

The present study aims to optimize the ethanol-reflux extraction conditions for extracting saponins from steamed *Panax notoginseng* (SPN). Four variables including the extraction time (0.5–2.5 h), ethanol concentration (50–90%), water to solid ratio (W/S, 8–16), and times of extraction (1–5) were investigated by using the Box-Behnken design response surface methodology (BBD-RSM). For each response, a second-order polynomial model with high *R*^2^ values (>0.9690) was developed using multiple linear regression analysis and the optimum conditions to maximize the yield (31.96%), content (70.49 mg/g), and antioxidant activity (EC_50_ value of 0.0421 mg/mL) for saponins extracted from SPN were obtained with a extraction time of 1.51 h, ethanol concentration of 60%, extraction done 3 times, and a W/S of 10. The experimental values were in good consistency with the predicted ones. In addition, the extracted SPN saponins could significantly increase the levels of blood routine parameters compared with the model group (*p* < 0.01) and there was no significant difference in the hematopoiesis effect between the SPN group and the SPN saponins group, of which the dose was 15 times lower than the former one. It is suggested that the SPN saponins extracted by the optimized method had similar functions of “blood tonifying” at a much lower dose.

## 1. Introduction

*Panax notoginseng* (PN) (Burk.) F. H. Chen, a highly valued Chinese medicinal herb, has been used in Asia to treat blood disorders for thousands of years [1]. Numerous studies have shown that saponins are the major active components of PN, with pharmacologic effects such as dilating blood vessels, lowering the blood pressure, anti-thrombosis, anti-inflammation, anti-vascular aging, anti-cancer, and antioxidant activities [2,3,4]. Therefore, a large number of studies have focused on the technology of extraction and purification of saponins. Related products with PN saponins as the main ingredients have even been developed. For example, Xuesaitong, one of the bestselling prescriptions of herbal medicine, consists of over 85% ginsenosides attributed to the extract obtained from PN, showing good therapeutic effects on the cardiovascular and cerebrovascular system, blood system, and nervous system [5].

There was a saying for PN that “the raw materials eliminate and the steamed ones tonify”. The so-called “eliminate” means raw PN can stop bleeding, promote blood circulation, diminish swelling, and ease pain [2]. The “tonify” means that steamed PN (SPN) performs better efficacies on improving the immunity and nourishing the blood [6,7]. The variation in the types and contents of saponins has been reported to be the reason resulting in the efficacy difference between raw and SPN [8,9,10]. According to our previous studies [11,12], the contents of notoginsenoside R_1_, ginsenosides Rg_1_, Rb_1_, Re, and Rd in raw PN were decreased along with the duration of steaming, whereas those of ginsenosides Rh_1_, Rk_3_, Rh_4_, 20(*R*)-Rg_3_, and 20(*S*)-Rg_3_ were increased, which were also found to be closely related to the tonifying functions of SPN. However, the current studies on the total saponins of PN were generally focused on those from raw materials and there were few reports on the processing of extracts or total saponins from SPN, not to speak of their activities, which hinders the development of this valuable medicine. Therefore, the ethanol-reflux extraction process of saponins from SPN was optimized in this research. Constituents of notoginsenoside R_1_, ginsenosides Rg_1_, Rb_1_, Re, Rd, Rh_1_, Rk_3_, Rh_4_, 20(*R*)-Rg_3_, 20(*S*)-Rg_3_ were included as the indices of saponins from SPN based on our previous study. The yield, content, and antioxidant activity of saponins from SPN were evaluated for the process optimization.

The Box–Behnken design response surface methodology (BBD-RSM) is a collection of mathematical and statistical techniques useful for establishing models which can be used to evaluate multiple parameters and their interactions with quantitative data, and effectively optimizing complex extraction procedures in a statistical way [13,14,15]. It reduces not only the number of experimental trials, the development time, and overall cost determines, but also the interactions among the independent variables [16,17,18,19]. Therefore, in this research, BBD-RSM was used to determine the optimal conditions of ethanol-reflux extraction of saponins from SPN. Besides, SPN is traditionally used as a tonic to enrich blood and tonify the body, which can improve the blood deficiency syndrome by increasing the production of various blood cells in anemic conditions [6,7]. Thus, to verify the pharmacologic activity of extracted saponins from SPN, we also investigated the hematopoiesis effect of SPN saponins on the levels of blood routine parameters in anemic mice induced by acetylphenylhydrazine (APH) and cyclophosphamide (CTX).

## 2. Results and Discussion

### 2.1. HPLC Analyses

**Figure 1:**
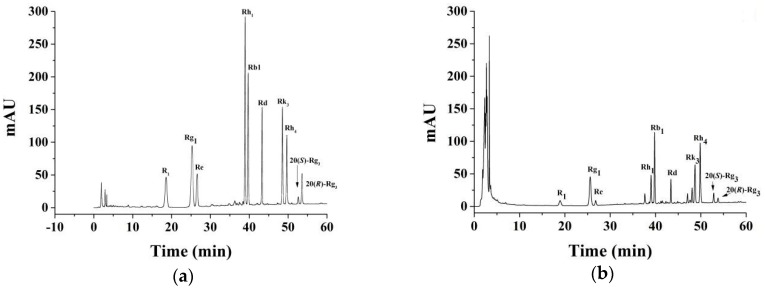
The HPLC chromatograms of the mixed standards solution (**a**) and SPN saponins (**b**). HPLC, high performance liquid chromatography; SPN, steamed *Panax notoginseng*.

The results of the HPLC analyses for the standards solution and sample solution were shown in Figure 1a,b. By comparing the chromatograms of SPN saponins to that of the mixed standards solution, constituents corresponding to major peaks were identified as notoginsenoside R_1_, ginsenosides Rg_1_, Re, Rb_1_, Rd, Rk_3_, Rh_4_, Rh_1_, 20(*S*)-Rg_3_, and 20(*R*)-Rg_3_. Among which, notoginsenoside R_1_, ginsenosides Rg_1_, and Rb_1_ were marker constituents for the quality control of PN in the Chinese Pharmacopoeia (2015 edition) [20]. Ginsenosides Re and Rd were active constituents related to the antioxidation effect of SPN [12]. The rest of the five ginsenosides were characteristic and active constituents of SPN, of which the contents were increased along with the increase of steaming temperature and duration of time. Therefore, the ten constituents were determined as the evaluation markers for the preparation of SPN saponins.

### 2.2. Single-Factor Experimental Analysis

#### 2.2.1. The Effect of Extraction Time on the Content and Yield of Saponins

**Figure 2:**
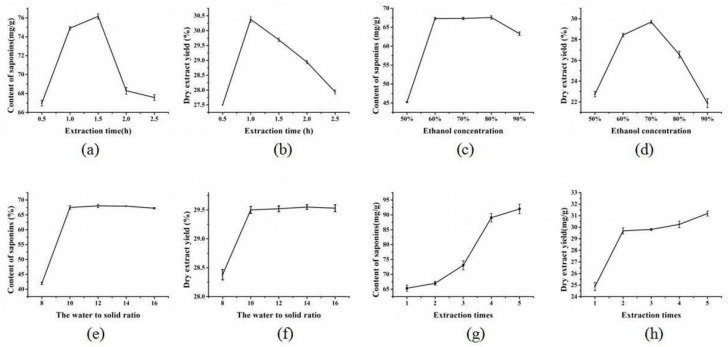
The result of the single-factor experimental analyses.

Extraction time is one of the most important factors that could affect the content of saponins and the yield. The content and yield of saponins under different extraction times are shown in Figure 2a,b. These results were obtained by, firstly setting the ethanol concentration, water to solid ratio, and extraction times to 70%, 10, and 2, respectively. The effect of extraction time on the content and yield of saponins was then investigated by sequentially setting the extraction time at 0.5, 1, 1.5, 2, and 2.5 h. According to the results, with the extension of extraction time, the content and yield of saponins both increased firstly and then decreased. The content of saponins and dry extract yield were firstly increased along with the extraction time, which took 1.5 h and 1 h to reach the maximum, respectively. After that, the levels of the two indexes decreased. The reason might be that at the initial stage of extraction, a relatively longer extraction time was beneficial for dissolving saponins and other polar compounds in the extract. However, if they were kept at high temperature for a long period of time, triterpenoids saponins could be easily decomposed [21]. The concentration of other ingredients in the extract reached the equilibrium with little change of dissolution and there might also be negative reactions causing the yield decline [22]. In addition, with the duration of steaming time, the increase of suspension viscosity was disadvantageous to the extraction efficiency [23] and some saponins might not be separated from the cell debris [24]. Based on the results, the extraction time of 1 h, 1.5 h, and 2 h could achieve saponins of higher content and yield. Therefore, 1 h, 1.5 h, and 2 h were selected as optimal conditions in the following BBD-RSM experiments.

#### 2.2.2. The Effect of Ethanol Concentration on the Content and Yield of Saponins

Another significant factor that would affect the content and yield of saponins is the ethanol concentration. Thus, in the present study, the effect of ethanol concentration on the content and yield of saponins was evaluated. The extraction time, water to solid ratio, and the times of extraction were set as 1.5, 10, and 2 h, respectively. The ethanol concentration was then sequentially set as 50%, 60%, 70%, 80%, and 90%. As shown in Figure 2c,d, when the ethanol concentration was varied between 50% and 90%, the content and yield of saponins increased firstly and then decreased. While the higher or lower ethanol concentration led to the decrease of the content and yield of saponins. A lower ethanol content means more water in the extract, which can result in the increased swelling of *Panax notoginseng*. Saponins can be extracted in a shorter time when the swelling of *Panax* *notoginseng* is greater. However, more saponins may hydrolyze when the water content in the mixed solvent is higher because of the higher boiling temperature [25]. According to the results, the ethanol concentrations of 60%, 70%, and 80%, were chosen to extract the saponins in the optimization step.

#### 2.2.3. The Effect of the Water to Solid Ratio on the Content and Yield of Saponins

Increasing the water to solid ratio can improve the content and yield of saponins by affecting the concentration gradient inside and outside cells in the extract. In the research, the water to solid ratios of 8, 10, 12, 14, and 16 were investigated. As shown in Figure 2e,f, as the water to solid ratio was increased from 8 to 10, the content and yield of saponins increased accordingly. This might be due to the increasing water to solid increasing the diffusivity of the solvent into the cells and enhancing the desorption of the saponins from the cells [26]. However, when the water to solid ratios were set at 10, 12, 14, and 16, the variation in the content and yield of saponins was not significant (*p* > 0.05). This might be due to the full dissolution of the components in the extract. Thus, in order to save the costs, the water to solid ratio was determined as 10.

#### 2.2.4. The Effect of the Times of Extraction on the Content and Yield of Saponins

Increasing the times of extraction can significantly improve the content of saponins and the dry extract yield. The effect of the times of extraction on the content and yield of saponins was shown in Figure 2g,h. The extraction time, ethanol concentration, and water to solid ratio were set as 1.5 h, 70%, and 10, respectively. The times of extraction was then sequentially set as 1, 2, 3, 4, and 5. It could be seen that with the increase of the times of extraction, the content and yield of saponins had different degrees of increase. At the same time, the production costs were constantly rising. Therefore, we selected extraction 2, 3, and 4 times as the optimal conditions in the following experiments.

In general, according to the single-factor experimental analysis, our study adopted the extraction time of 1 h, 1.5 h, and 2 h; the ethanol concentration of 60%, 70%, and 80%; the water to solid ratio of 10; and the times of extraction of 2, 3, and 4 for the BBD-RSM.

### 2.3. Fitting the Response Surface Models

As shown in Table 1 and Table 2, in the BBD-RSM experimental design, a total of 17 tests of different conditions were performed and the corresponding result of each test was also shown in Table 2. Meanwhile, the contents of ten saponins were shown in Table 3. According to the experimental data, the multiple linear regression equation between the response value and the experimental condition was calculated by using Design-Expert, version 8.6 (Stat-Ease Inc., Minneapolis, MN, USA). At the same time, the regression coefficients for each value were also determined. The fitted equations to predict the yield, content, and antioxidant activity of saponins from the SPN are given below regardless of the significance of the coefficients:

Yield of saponins = 31.13 + 0.52*X*_1_ − 1.36*X*_2_ + 0.81*X*_3_ + 0.45*X*_12_ − 0.5*X*_12_ − 0.54*X*_22_ − 0.52*X*_32_ (1)

Content of saponins = 70.14 − 1.65*X*_1_ + 6.09*X*_3_ − 1.91*X*_12_ − 1.50*X*_23_ − 2.32*X*_12_ − 2.97*X*_32_ (2)

EC_50_ = 0.042 + (5.713E − 003)*X*_1_ − 0.020*X*_3_ + (3.580E − 003)*X*_12_ + (4.550E − 003)*X*_23_ + (7.154E − 003)*X*_12_ + 0.012*X*_32_ (3)

**Figure 3:**
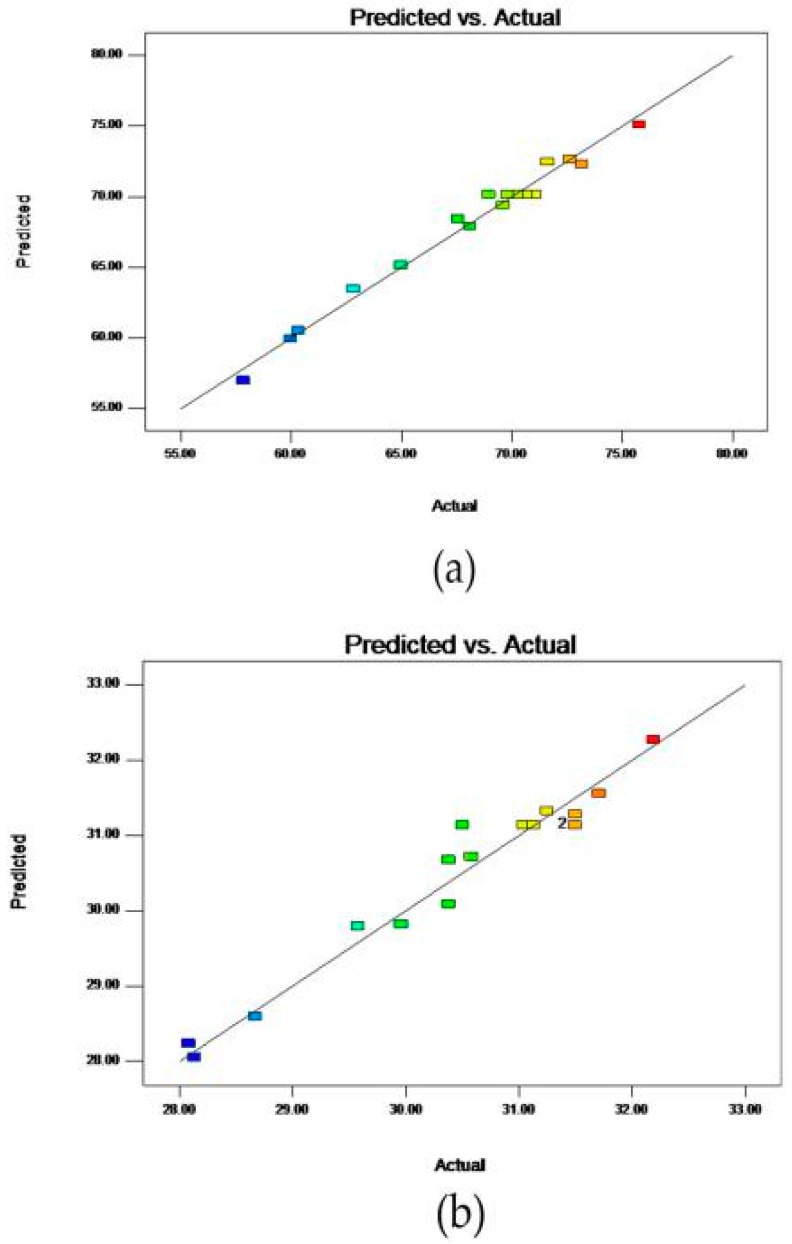

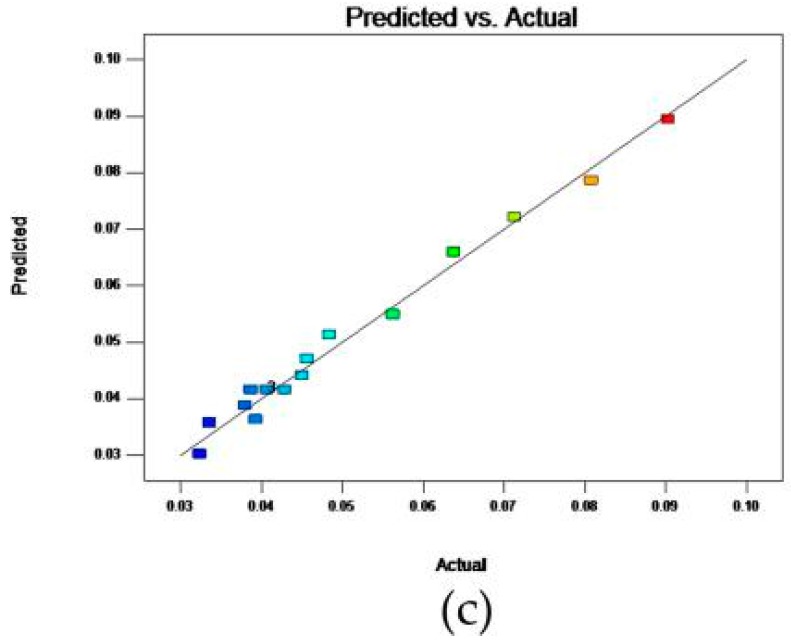
The comparison between the predicted and measured values of the content (**a**), yield (**b**), and antioxidant activity (**c**) of the saponins from the SPN.

The analysis of variance (ANOVA) was used to evaluate the significance of the quadratic polynomial models [27]. For each term in the models, a large *F*-value and a small *P*-value would imply a more significant effect on the respective response variable [28]. The ANOVA results of some important terms in the models were summarized in Table 4, Table 5 and Table 6. To verify the adequacy of a model, the coefficient of determination (*R*^2^), lack of fit, *R*^2^_adj_, AP, and CV tests were typically used. *R*^2^ represents a percentage of the variables that can be explained by the model. Commonly, the higher *R*^2^ not only represents the majority of the variables that can be explained by the model, but also represents that the experimental data are very consistent with the second-order polynomial equation. As shown in Table 4, Table 5 and Table 6, the *R*^2^ values were 0.9690, 0.9840, and 0.9874, respectively. This indicated that there was only 1.60–3.10% of the total variation which was not explained by the models. In these models, the values of *R*^2^ were high, which met our requirements. However, *R*^2^ is not a decisive factor and *R*^2^_adj_ is also important. *R*^2^_adj_ is a modification of *R*^2^ that adjusts for the number of explanatory terms in a model. Unlike *R*^2^, the *R*^2^_adj_ increases only if the new term improves the model more than would be expected by chance [29]. For the values of *R*^2^ and *R*^2^_adj_, the greater the better, the closer the better. From Table 3, Table 4 and Table 5, the values of *R*^2^_adj_ of the models were 0.9292, 0.9635, and 0.9713, respectively. The high values of *R*^2^_adj_ indicated that the model was significant. The significance of the lack of fit test indicated that the points were not properly distributed around the model; as a result, the model could not be applied to predict the values of the independent variables. Therefore, the insignificance of the lack of fit test implied that the model was able to fit the data properly [13]. In our models, all the values of “*p*-value prob > *F*” of the lack of fit were greater than 10% and they were insignificant. This indicated that the model was able to fit the data properly. “AP” measures the signal to noise ratio. A ratio greater than 4 is desirable. In these models, the values of AP were 16.965, 24.331, and 27.165, respectively. This indicated an adequate signal. As a general rule, the CV should not be greater than 10% [30]. The values of the CV of the models were 1.16, 1.43, and 5.67, respectively. In addition to the above important parameters which could verify the suitability of the model, the figure of the predicted value versus the measured one can also be used to prove it. As shown in Figure 3, the predicted value of the model and the actual value of the experiment were fitted almost in a straight line. It proved that the second-order polynomial regression model was in good agreement with the experimental results and indicated that the models applied in this study were able to identify the operating conditions for selective extraction of saponins from SPN.

### 2.4. Analysis of Influence of Variables on the Yield of Saponins

**Figure 4:**
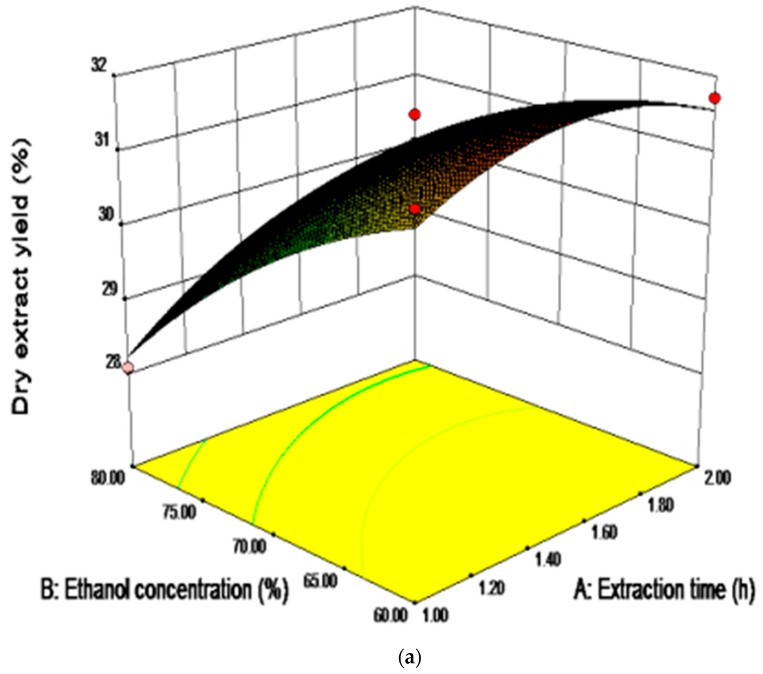

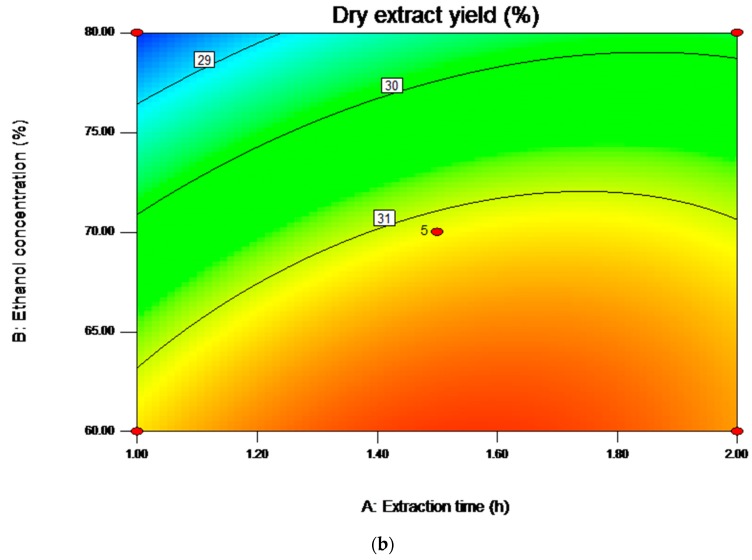
The response surface (**a**) and contour (**b**) plots showing the significant (*p* < 0.05) interaction effects of ethanol concentration with extraction time on the yield of saponins.

In Table 4, the liner and quadratic effects of the extraction time, ethanol concentration, and times of extraction were significant (*p* < 0.05). The most significant effect on the yield of saponins was shown to be the linear effect of ethanol concentration (*p* < 0.05). Among the different interaction effects, there was only one interaction of extraction time with ethanol concentration which was significant (*p* < 0.05). Figure 4a presented the interaction between the extraction time and ethanol concentration. The yield of saponins was initially increased along with the duration of the extraction, following the decrease which might be due to the providence of the time requirement of the exposure of the ingredients of SPN to the release medium where the liquid penetrated into the cell wall of dried raw materials, dissolved the saponins, and subsequently diffused out from the raw materials [31]. However, with the extension of the extraction time, the components in the solvent were transformed and even degraded. At the same time, the yield of the saponins was significantly decreased by increasing the ethanol concentration, which was probably due to the decreased solubility of the weakly-polar and non-polar components, such as carbohydrates contained in SPN, induced by the increase of the solvent polarity. As shown in Figure 4a,b, the yield of the saponins reached the maximum when the extraction time and ethanol concentration were approximately 1.56 h and 60%, respectively.

### 2.5. Analysis of Influence of Variables on the Content of Saponins

From Table 5, the linear and quadratic effects of the extraction time and extraction times were found to be significant on the content of saponins extracted from SPN (*p* < 0.01). However, the linear and quadratic effects of the ethanol concentration were not significant (*p* > 0.05). Among the different interaction effects, only the interactions of extraction time with ethanol concentration, and ethanol concentration with extraction times were significant (*p* < 0.05). As shown in Table 5, the most significant (*p* < 0.05) effect on the content of saponins was shown to be the linear one of the extraction times followed by the quadratic effect of extraction time. 

**Figure 5:**
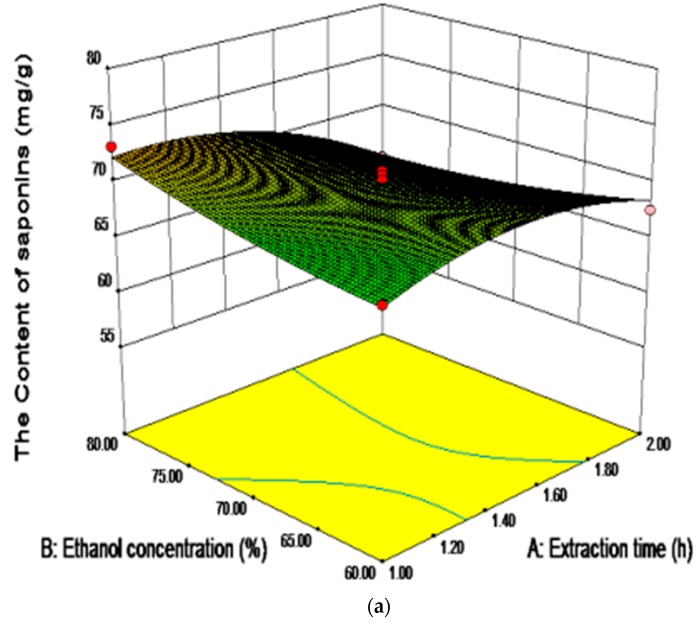

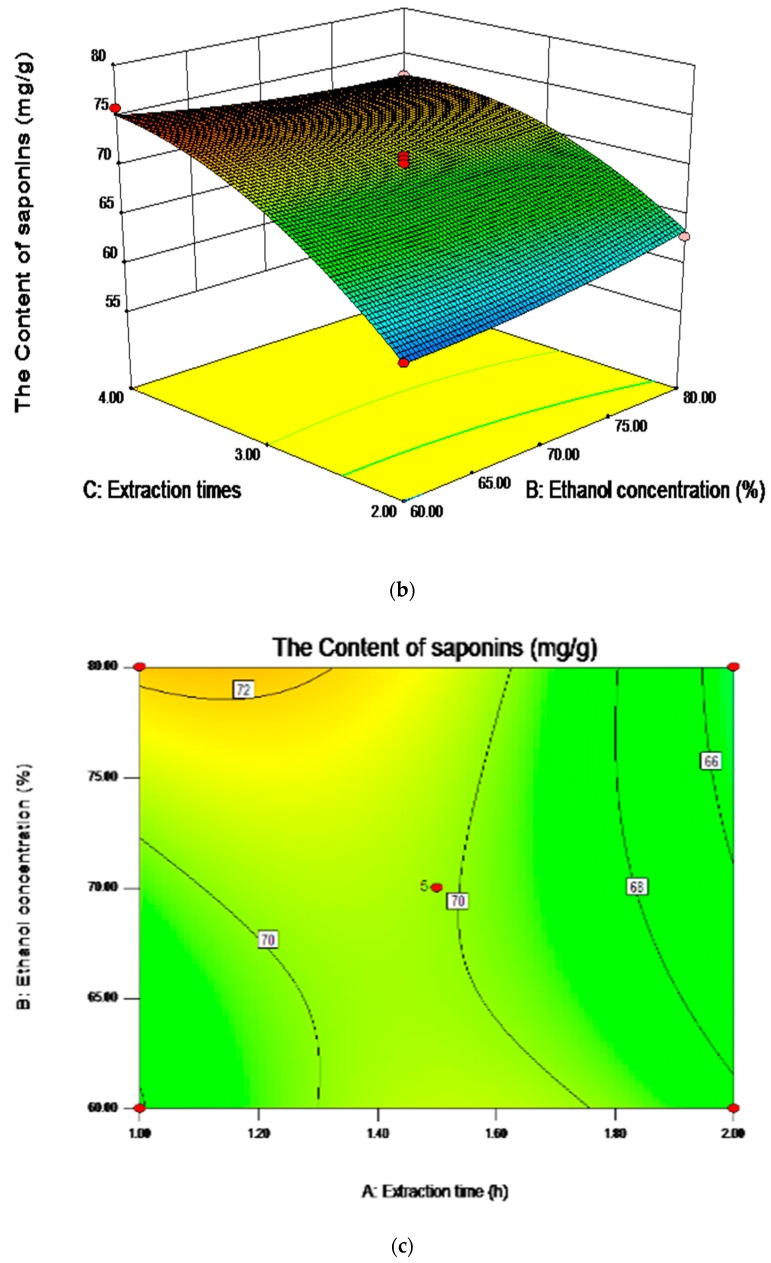

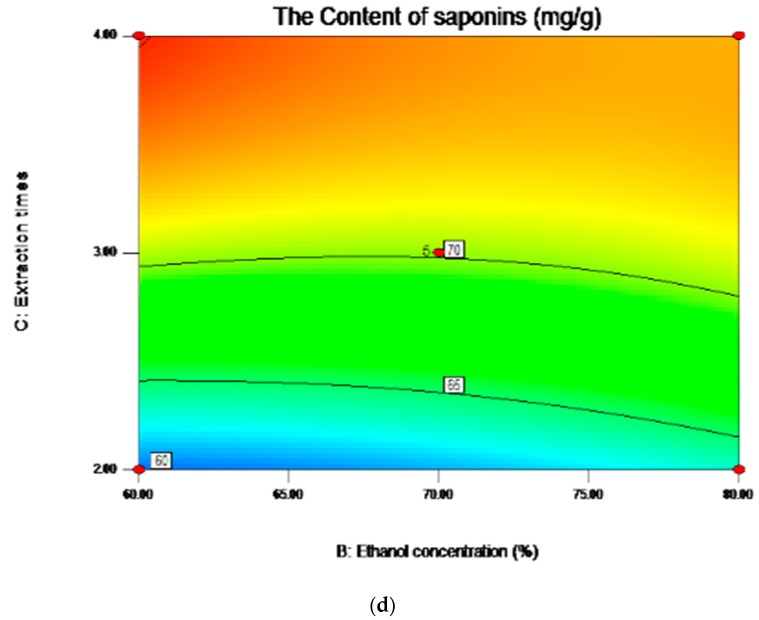
The response surface (**a**,**b**) and contour (**c**,**d**) plots showing the significant (*p* < 0.05) interaction effects of the ethanol concentration with the extraction time and extraction times on the content of saponins.

Figure 5a presents the interaction between the extraction time and ethanol concentration. Initially, the content of saponins was increased along with the duration of the extraction time, then following the decrease which might be due to the time requirement of the exposure of the saponins from the SPN to the release medium. However, with the extension of the extraction time, the saponins in the solvent were transformed and even degraded. As shown in Figure 5a,c, the content of saponins reached the maximum when the extraction time and ethanol concentration were approximately 1.50 h and 70%, respectively.

Figure 5b shows the interaction between the times of extraction and ethanol concentration. The content of saponins was significantly increased with the increase of the times of extraction, which might be due to the full degradation of saponins from SPN. As shown in Figure 5b,d, the content of saponins reached the maximum when the times of extraction and ethanol concentration were approximately 3% and 70%, respectively.

### 2.6. Analysis of Influence of Variables on the Antioxidant Activity

In our study, the effect of the ethanol-reflux extraction variables on the antioxidant activity of saponins from SPN was determined based on the hydroxyl radicals scavenging activity because, in our previous study, we found that SPN exhibited a stronger activity of scavenging hydroxyl radicals than scavenging DPPH free radicals [11]. Hydroxyl radicals are strong free radicals. The excessive hydroxyl radicals have a close relationship with various diseases and health problems, such as aging arthritis, cancer, inflammation, and heart diseases [32]. In the research, the antioxidant activity of SPN saponins was evaluated by determining the EC_50_ value of the hydroxyl radical scavenging capacity. The lower value of EC_50_ indicated a stronger clearance ability.

**Figure 6:**
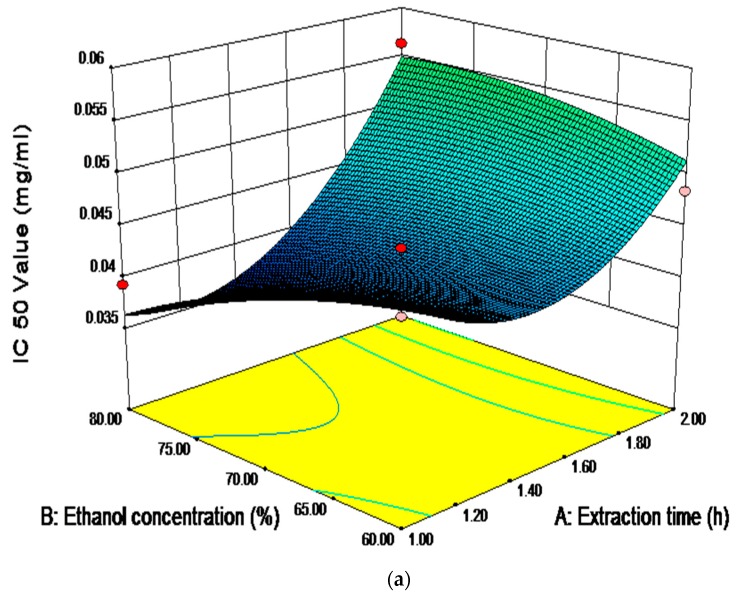

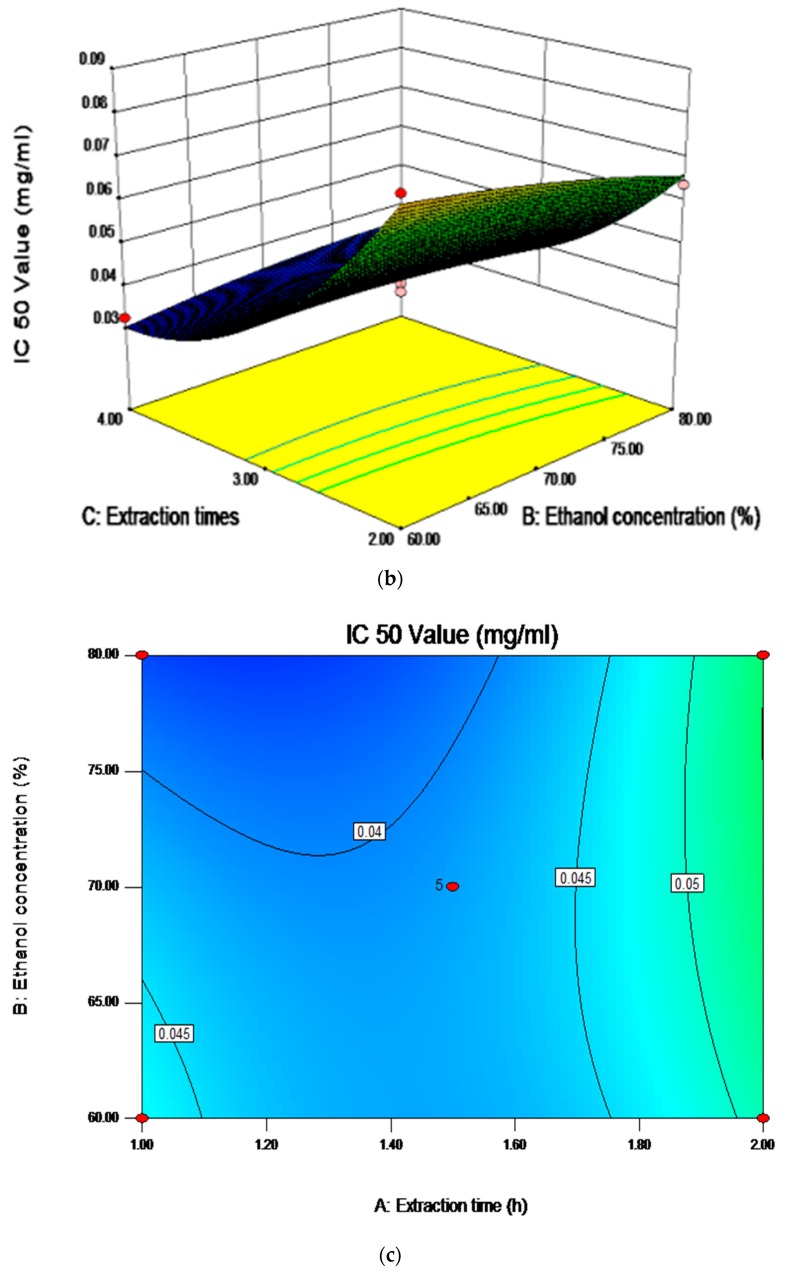

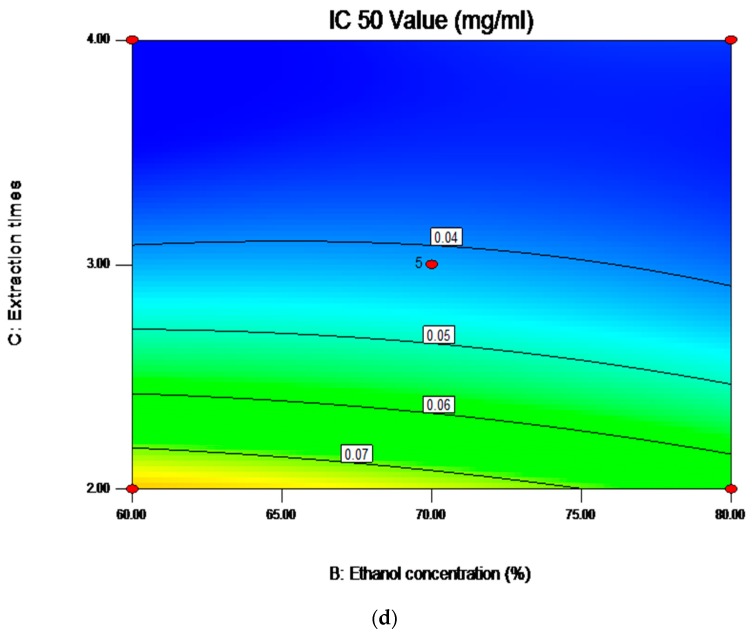
Response surface (**a**,**b**) and contour (**c**,**d**) plots showing the significant (*p* < 0.05) interaction effects of ethanol concentration with extraction time and extraction times on the value of EC _50_.

According to Table 6, the linear and quadratic effects of the extraction time and times of extraction on the hydroxyl radicals scavenging activity were significant (*p* < 0.05). However, the linear and quadratic effects of the ethanol concentration on the antioxidation were not significant (*p* > 0.05). Among different interaction effects, the interactions of the extraction time with ethanol concentration, and the ethanol concentration with the times of extraction were significant (*p* < 0.05). As shown in Figure 6a,b, the EC_50_ value decreased along with the increase of the extraction time and times of extraction. The results indicated that the EC_50_ value was minimized when the extraction time and times of extraction were approximately 1.5 h and 3 times, respectively.

### 2.7. Optimization and Validation Procedures

To maximize the yield, content, and antioxidant activity of SPN saponins, the quadratic models within the studied experimental range of various process variables were established. The predicted optimal conditions were shown as follows: the extraction time of 1.51 h, ethanol concentration of 60%, and extraction times of 3. Under the recommended optimum extraction condition, the predicted values of the yield, the content of saponins, and the EC_50_ of antioxidant activity were 31.95%, 70.49 mg/g, and 0.0421 mg/mL, respectively. The corresponding experimental values of the above indices were determined as 31.94 ± 0.02%, 70.46 ± 0.0971 mg/g, and 0.0419 ± 0.0005 mg/mL, respectively, which were very close to the values predicted by the constructed models. In addition, compared with our previous study [12] on the antioxidant activity of SPN (EC_50_ value of 1.197 ± 0.11 mg/mL), the EC_50_ value of 0.0419 ± 0.005 mg/mL of SPN saponins was significantly lower, indicating that the antioxidant activity of SPN saponins extracted by the optimized method was much stronger than the unprocessed SPN powder.

### 2.8. Blood Routine Test

**Figure 7:**
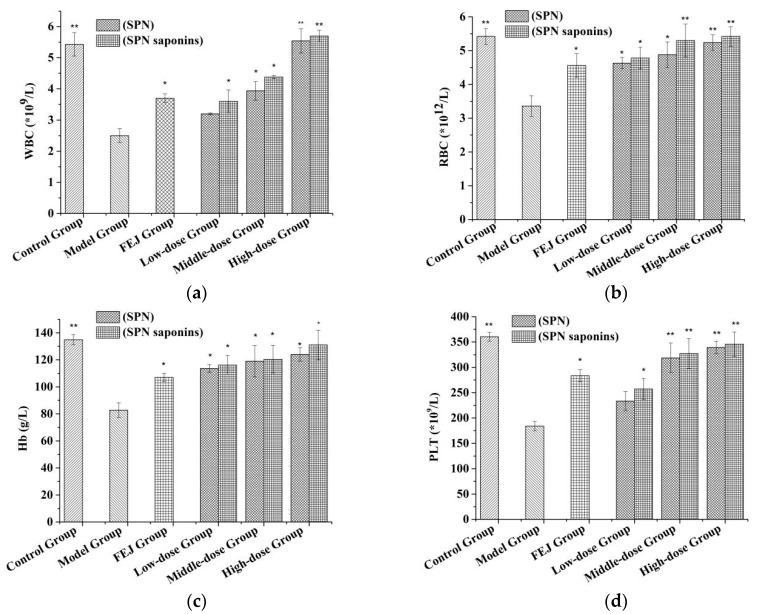
The blood parameters after treating SPN and SPN saponins, where (**a**) is the content of WBC, (**b**) is the content of RBC, (**c**) is the content of Hb and (**d**) is the content of platelet. Each value represents means ± SD (*n* = 10); *p < 0.05 and ** *p* < 0.01 compared to the control group.

After the administration for 12 days, the quantities of white blood cell (WBC), red blood cell (RBC), platelet (PLT) and hemoglobin (Hb) from the peripheral blood of mice were shown in Figure 7. Compared with the control group, the levels of WBC, RBC, PLT, and Hb in the model group were significantly decreased (*p* < 0.01), indicating the anemia model was successfully established. Compared with the model group, WBC, RBC, PLT, and Hb levels in the *Fufang E’jiao Jiang* (FEJ) and three doses of SPN and SPN saponins groups were increased at different degrees. Besides, the levels of the above four parameters were increased with the added dose of SPN and SPN saponins in a dose-dependent manner. For the same type of dose, there was no significant difference in the hematopoiesis effect between SPN and SPN saponins, although the dose of SPN saponins was 15 times lower than the former one. It suggested that taking a much smaller amount of SPN saponins could achieve the similar hematopoiesis effect as SPN. 

Traditionally, SPN is used as a tonic to enrich blood and tonify the body, which can improve anemia by increasing the production of various blood cells in anemic conditions [6,7]. Besides, the body tonifying function of herbal medicines is partly attributed to their antioxidant effect by modern pharmacological research [33], which can be evaluated by investigating the hydroxyl radical scavenging activity [32]. Therefore, the changed levels of blood parameters and EC_50_ value of the hydroxyl radical scavenging activity were determined to evaluate the tonifying efficacies of SPN. The lower the value of EC_50_, the stronger the antioxidant ability. According to the results, the crude saponins of SPN obtained by the optimized extraction method did not only exhibit strong antioxidant activity, but also show good hematopoiesis effect on increasing the levels of blood parameters in a dose-dependent way. The consistency in improving the functions of antioxidation in vitro and hematopoiesis in vivo suggested that the crude extract of saponins with strong activities related to the clinic efficacies of SPN could be obtained by the optimized extraction method.

## 3. Materials and Methods

### 3.1. Plant Material and Chemicals

SPN samples were prepared by steaming the crushed raw PN in an autoclave (Shanghai, China) at 120 °C for 2 h. The steamed powder was then dried in a heating-air drying oven at about 45 °C until constant weight and then sieved through a 40 mesh sieve. Ethanol was of analytical grade and purchased from Tianjin Feng Chuan Chemical Reagent Technologies Co, Ltd. (Tianjin, China). Acetonitrile of chromatographic grade and ferrous chloride and hydrogen peroxide of analytical grade were purchased from Merck Chemical Co. (Darmstadt, Germany). Notoginsenoside R_1_, ginsenosides Rg_1_, Re, Rb_1_, Rd, Rh_1_, Rk_3_, Rh_4_, 20(*S*)-Rg_3_, and 20(*R*)-Rg_3_ (Sichuan Weikeqi Biological Technology Co., Ltd. Chengdu, China) with a purity ≥ 98% were used as the standard compounds.

### 3.2. Animals 

Animal experimental procedures in the study strictly conformed to the Guide for the Care and Use of Laboratory Animals and related ethics regulations of Kunming University of Science and Technology. The protocol was approved by the Experimental Animal Welfare and Ethics Committee, Kunming University of Science and Technology (project number: 81660661; code: KKGD201626039; date of approval: 9 January 2017). The experimental method refers to our previous study [11], in which “Kunming mice, male and female, weighing 18–22 g, were purchased from Tianqin Biotechnology Co., Ltd., Changsha, China [SCXK (Xiang) 2014-0011]. Before the experiments, the mice were given a one-week acclimation period in a laboratory at room temperature (20–25 °C) and constant humidity (40–70%), and fed with standard rodent chow and tap water freely.”

### 3.3. Ethanol-Reflux Extraction Process

In order to obtain saponins from SPN, ethanol-reflux extraction was used. Ethanol-reflux extraction was performed by using ethanol as the extraction solvent at the given extraction time, ethanol concentration, water to solid ratio, and times of extraction. That is to say, a 5.0 g sample powder was extracted by 50 mL of 70% ethanol at 85 °C in a water bath for 1.5 h. After extraction three times, the extraction of ethanol-reflux was combined. Subsequently, the extract solution was centrifuged, filtered, concentrated, and dried to obtain the crude saponins of SPN.

### 3.4. Determination of the Yield of Saponins.

The saponin extraction yield (%) was obtained by dividing the dried crude saponins weight (g) to powder weight (g) and it was calculated by the following equation:

Yield (%) = dried crude saponins weight (g)/powder weight (g) × 100%(4)

### 3.5. Determination of the Content of Saponins

HPLC analyses were performed according to the previous method [11]. The crude SPN saponins were dissolved with 20 mL of ultra-pure water. The supernatant after the filtration of the solution was used as the sample solution. “A mixed standards solution containing (in mg/mL) 0.40 notoginsenoside R_1_, 0.55 ginsenosides Rg_1_, 0.50 Re, 0.60 Rb_1_, 0.50 Rd, 0.60 Rh_1_, 1.00 Rk_3_, 1.00 Rh_4_, 0.45 20(*S*)-Rg_3_ and 0.55 20(*R*)-Rg_3_, was prepared by adding each standard into a volumetric flask and dissolving with methanol. A series of standards solutions of seven concentrations were prepared by diluting the mixed standard solution with methanol for the determination of the standard curves. HPLC analyses were done on a 1260 series system (Agilent Technologies, Santa Clara, CA, USA) consisting of a G1311B Pump, a G4212B DAD detector, and a G1329B autosampler. A Vision HT C_18_ column (250 mm × 4.6 mm, 5 μm) (welch Co., Ltd., Shanghai, China) was adopted for the analyses. The mobile phase was comprised of A (ultra-pure water) and B (methyl cyanide). The gradient mode was as follows: 0–20 min, 80% A; 20–45 min, 54% A; 45–55 min, 45% A; 55–60 min, 45% A; 60–65 min, 100% B; 65–70 min, 80% A; 70–90 min, 80% A. The flow rate was set at 1.0 mL/min. The detection wavelength was set at 203 nm. The column temperature was set at 30 °C and the sample volume was set at 10 μL.” 

### 3.6. Measurement of Antioxidant Activity

The antioxidant activity was determined based on the hydroxyl radicals scavenging activity. The scavenging activity for hydroxyl radicals was measured with the Fenton reaction [34]. The reaction mixture contained 60 μL of 1.0 mM FeCl_2_, 90 μL of 1 mM 1,10-phenanthroline, 2.4 mL of 0.2 M phosphate buffer (pH 7.8), 150 μL of 0.17 M H_2_O_2_, and 1.5 mL of extracts at various concentrations. The reaction was started by adding H_2_O_2_. After incubation at room temperature for 5 min, the absorbance of the mixture at 560 nm was measured with a Beckman spectrophotometer(Shimadzu corporation, kyoyo, Japan). The hydroxyl radicals scavenging activity was calculated according to the following equation:

Scavenging rate = [1 − (*A*_1_ − *A*_2_)/*A*_0_] × 100% (5)

where *A*_0_ was the absorbance of the control (blank, without extract), *A*_1_ was the absorbance in the presence of the extract, and *A*_2_ was the absorbance without 1,10-phenanthroline [32].

### 3.7. BBD-RSM Experimental Design

BBD-RSM was used to optimize the effect of the extraction parameters including the extraction time (1–2 h), ethanol concentration (60–80%), and times of extraction (2–4) on the yield, content, and antioxidant activity of the saponins from the SPN. Based on the preliminary range of the process variables in a single factor test, the BBD-RSM with three mentioned independent variables at three levels was carried out (Table 1 and Table 2). In this design, the randomized run order was created by considering six factorial points, six axial points, and five center points. For the model analysis, the results of the experimental design were fitted by a polynomial equation to correlate the response to the independent variables. The general equation to predict the optimal point was explained as follows:

*Y* = b_0_ + b_1_*X*_1_ + b_2_*X*_2_ + b_3_*X*_3_ + b_11_*X*_11_ + b_22_*X*_22_ + b_33_*X*_33_ + b_12_*X*_12_ + b_13_*X*_13_ + b_23_*X*_23_ (6)

where *Y* is the predicted response; *b*_0_, *b*_1_, *b*_2_, *b*_3_, *b*_11_, *b*_22_, *b*_33_, *b*_12_, *b*_13_, and *b*_23_ represent the regression coefficients; and *X*_1_, *X*_2_, *X*_3_, *X*_4_ are the coded independent factors. The regression coefficient (*R*^2^), adjusted-*R*^2^ (*R*^2^_adj_), the prediction error sum of squares (PRESS), and adequate precision (AP) were used to determine the goodness-of-fit of the constructed polynomial models.

### 3.8. Blood Routine Test

One hundred and eight Kunming mice, half male and half female, were randomly divided into nine groups, namely the control group, model group, FEJ group, high, moderate, low-dose SPN groups, and the high, moderate, and low-dose SPN saponins groups; 12 mice in each group. The APH and CTX-induced anemia model was applied to evaluate the “blood tonifying” function of SPN saponins combined with previous methods [35]. The anemia model was established by intraperitoneal injection of CTX of 0.07 g/kg for the first three days and a hypodermic injection of APH of 0.02 g/kg on the fourth day. Mice in the control group were administered with 0.9% normal saline, whereas the other groups were administered with FEJ (8 g/kg), SPN (0.45 g/kg, 0.90 g/kg, and 1.8 g/kg, respectively), and SPN saponins (0.03 g/kg, 0.06 g/kg, and 0.12 g/kg, respectively), respectively, by gavage for 12 days. After 30 minutes of the last administration, the blood was collected from the tail vein of mice and then removed from a centrifuge tube containing heparin sodium. Then the collected blood was used for the routing analysis by an automatic blood analyzer (Healife, Taian, China), including levels of WBC, RBC, Hb, and PLT after 30 min of the last administration.

### 3.9. Statistical Analysis

The software Design-Expert (version 8.6.0, Stat-Ease Inc., Minneapolis, USA) was used to calculate the relationship between the independent variables and responses. And all the data were expressed as means ± SD. The SPSS 21.0 software (Statistical Program for Social Sciences, SPSS Inc., Chicago, IL, USA) was applied to carry out the two-tailed unpaired t-test. A value of *p* < 0.05 was considered to be a significant difference. A value of *p* < 0.01 was considered to be a highly significant difference. The EC_50_ value was fitted by probit regression with the Origin 7.5 software for Windows (OriginLab Corporation, Northampton, MA, USA).

## 4. Conclusions

In the research, the effects of different ethanol-reflux extraction variables (extraction time, ethanol concentration, the water to solid ratio, and extraction times) on the yield, content, and antioxidant activity of saponins from SPN were studied and the extraction condition to obtain saponins from SPNs was successfully optimized by using BBD-RSM. The second-order polynomial models for all the response variables were found to be statistically significant. The optimal extraction conditions were as follows: an extraction time of 1.51 h, an ethanol concentration of 60%, and extraction 3 times. Under this condition, the yield, content, and EC_50_ of antioxidant activity of SPN saponins were 31.94 ± 0.02%, 70.46 ± 0.0971 mg/g, and 0.0419 ± 0.0005 mg/mL, respectively, which were corresponding well with the predicted values (31.95%, 70.49 mg/g, and 0.0421 mg/mL) by the models. Compared with the unprocessed SPN powder, the extracted SPN saponins also showed significantly stronger antioxidant activity and similar hematopoiesis effects at a 15-times lower concentration level, indicating that the optimized extracted method could be beneficial for producing SPN saponins with good therapeutic functions.

## Figures and Tables

**Table 1:** The experimental domain of BBD-RSM. BBD-RSM, Box-Behnken design response surface methodology.

Independent Variables	Unit	Symbol	Coded Levels		
			–1	0	+1
Extraction time	min	*X_1_*	60	90	120
Ethanol concentration	%	*X_2_*	60	70	80
Times of extraction		*X_3_*	2	3	4

**Table 2:** The BBD matrix and the experimental data for the responses. BBD-RSM, Box-Behnken design response surface methodology.

Treatment Number	Extraction Time (h)	Ethanol Concentration (%)	Times of Extraction	Content of Saponins (mg/g)	Dry Extract Yield (%)	EC_50_ Value (mg/mL)
1	1.00	80.00	3.00	73.16 ± 0.058	28.08 ± 0.038	0.0393 ± 0.0006
2	1.50	80.00	2.00	62.81 ± 0.069	28.13 ± 0.026	0.0638 ± 0.0005
3	1.50	70.00	3.00	68.96 ± 0.072	31.13 ± 0.019	0.0407 ± 0.0008
4	1.50	70.00	3.00	69.81 ± 0.135	31.50 ± 0.0017	0.0429 ± 0.0006
5	2.00	80.00	3.00	64.97 ± 0.046	29.58 ± 0.023	0.0563 ± 0.0005
6	2.00	70.00	2.00	57.83 ± 0.083	29.96 ± 0.026	0.0903 ± 0.0009
7	1.50	70.00	3.00	71.05 ± 0.122	31.04 ± 0.057	0.0429 ± 0.0004
8	2.00	60.00	3.00	67.55 ± 0.096	31.71 ± 0.049	0.0484 ± 0.0007
9	1.50	60.00	4.00	75.76 ± 0.073	32.19 ± 0.025	0.0324 ± 0.0005
10	1.00	70.00	4.00	71.62 ± 0.108	30.58 ± 0.027	0.0380 ± 0.0005
11	1.50	80.00	4.00	72.62 ± 0.125	30.38 ± 0.018	0.0336 ± 0.0006
12	1.00	60.00	3.00	68.08 ± 0.098	31.49 ± 0.043	0.0457 ± 0.0008
13	2.00	70.00	4.00	69.6 ± 0.062	31.25 ± 0.025	0.0450 ± 0.0009
14	1.00	70.00	2.00	60.32 ± 0.085	28.67 ± 0.036	0.0713 ± 0.0011
15	1.50	70.00	3.00	70.65 ± 0.094	31.50 ± 0.054	0.0429 ± 0.0006
16	1.50	60.00	2.00	59.96 ± 0.067	30.38 ± 0.075	0.0808 ± 0.0012
17	1.50	70.00	3.00	70.22 ± 0.083	30.50 ± 0.026	0.0387 ± 0.0008

**Table 3:** The contents of the ten markers for SPN saponins.

Treatment Number	R_1_ (mg/g)	Rg_1_ (mg)	Rb_1_ (mg/g)	Re (mg/g)	Rd (mg/g)	Rh_1_ (mg/g)	Rk_3_ (mg/g)	Rh_4_ (mg/g)	20(*R*)-Rg_3_ (mg/g)	20(*S*)-Rg_3_ (mg/g)
1	4.71 ± 0.075	18.51 ± 0.163	17.29 ± 0.132	2.57 ± 0.036	6.76 ± 0.079	0.12 ± 0.025	6.78 ± 0.098	11.41 ± 0.125	1.87 ± 0.028	3.14 ± 0.053
2	3.72 ± 0.036	14.53 ± 0.172	15.54 ± 0.195	1.92 ± 0.063	5.63 ± 0.138	0.05 ± 0.009	6.70 ± 0.084	9.50 ± 0.112	2.87 ± 0.096	2.35 ± 0.107
3	5.25 ± 0.052	21.52 ± 0.164	17.94 ± 0.118	3.30 ± 0.082	6.55 ± 0.125	0.15 ± 0.019	4.02 ± 0.043	7.64 ± 0.124	0.77 ± 0.086	1.84 ± 0.097
4	5.55 ± 0.068	21.79 ± 0.155	17.41 ± 0.184	3.11 ± 0.065	5.78 ± 0.153	0.14 ± 0.008	4.57 ± 0.052	7.61 ± 0.098	1.34 ± 0.052	2.53 ± 0.025
5	3.91 ± 0.034	17.17 ± 0.125	16.28 ± 0.185	2.28 ± 0.083	5.64 ± 0.126	0.11 ± 0.007	5.69 ± 0.086	9.76 ± 0.153	1.47 ± 0.036	2.65 ± 0.026
6	1.12 ± 0.025	10.52 ± 0.126	14.09 ± 0.208	1.69 ± 0.057	5.17 ± 0.134	0.03 ± 0.002	6.85 ± 0.068	12.53 ± 0.209	2.39 ± 0.058	3.43 ± 0.053
7	5.46 ± 0.086	23.35 ± 0.126	18.85 ± 0.152	3.43 ± 0.084	6.10 ± 0.275	0.12 ± 0.011	4.12 ± 0.045	6.86 ± 0.095	0.83 ± 0.063	1.93 ± 0.087
8	4.83 ± 0.047	19.78 ± 0.165	15.98 ± 0.126	3.16 ± 0.096	5.15 ± 0.159	0.13 ± 0.023	5.40 ± 0.058	9.36 ± 0.089	1.27 ± 0.086	2.48 ± 0.082
9	5.63 ± 0.058	20.62 ± 0.396	18.05 ± 0.151	3.15 ± 0.113	6.72 ± 0.223	0.13 ± 0.008	6.25 ± 0.066	10.85 ± 0.103	1.58 ± 0.032	2.78 ± 0.096
10	4.85 ± 0.063	18.99 ± 0.154	17.51 ± 0.185	3.06 ± 0.079	6.53 ± 0.128	0.12 ± 0.007	6.04 ± 0.059	10.54 ± 0.108	1.25 ± 0.025	2.71 ± 0.057
11	4.15 ± 0.035	21.09 ± 0.157	17.87 ± 0.182	3.30 ± 0.102	5.56 ± 0.135	0.16 ± 0.005	5.88 ± 0.048	10.23 ± 0.099	1.53 ± 0.041	2.85 ± 0.035
12	4.93 ± 0.028	20.19 ± 0.065	17.37 ± 0.093	2.52 ± 0.094	5.76 ± 0.124	0.13 ± 0.009	5.00 ± 0.038	8.64 ± 0.075	1.22 ± 0.086	2.32 ± 0.026
13	5.06 ± 0.096	21.69 ± 0.182	17.87 ± 0.133	3.32 ± 0.087	5.84 ± 0.119	0.12 ± 0.006	4.73 ± 0.086	7.98 ± 0.082	0.94 ± 0.023	2.05 ± 0.345
14	2.22 ± 0.025	11.19 ± 0.168	14.28 ± 0.182	1.85 ± 0.058	5.25 ± 0.132	0.06 ± 0.002	6.93 ± 0.073	12.63 ± 0.096	2.45 ± 0.055	3.47 ± 0.182
15	5.50 ± 0.125	23.04 ± 0.154	18.40 ± 0.121	3.37 ± 0.106	6.48 ± 0.157	0.12 ± 0.011	4.17 ± 0.021	6.82 ± 0.135	0.81 ± 0.062	1.92 ± 0.069
16	1.90 ± 0.019	10.94 ± 0.096	14.60 ± 0.086	1.13 ± 0.048	5.15 ± 0.122	0.04 ± 0.002	7.27 ± 0.102	13.08 ± 0.152	2.39 ± 0.096	3.45 ± 0.122
17	5.15 ± 0.097	21.82 ± 0.193	18.14 ± 0.063	3.31 ± 0.115	5.90 ± 0.112	0.11 ± 0.005	4.65 ± 0.083	7.81 ± 0.096	0.96 ± 0.025	2.06 ± 0.083

**Table 4:** The ANOVA results for the response surface quadratic models for the yield of saponins extracted from the SPN.

Source (Yield of Saponins)	Sum of Squares	DF	Mean Square	*F* Value	*p-*Value
Model	27.03	9	3.00	24.33	0.0002
*X*_1_	2.17	1	2.17	17.61	0.0041
*X*_2_	14.88	1	14.88	120.54	<0.0001
*X*_3_	5.22	1	5.22	42.26	0.0003
*X*_12_	0.80	1	6.49	6.49	0.0382
*X*_13_	0.096	1	0.78	0.78	0.4060
*X*_23_	0.032	1	0.26	0.26	0.6242
*X*_12_	1.05	1	8.47	8.47	0.0227
*X*_22_	1.24	1	10.07	10.07	0.0156
*X*_32_	1.14	1	9.25	9.25	0.0188
Residual	0.86	7	0.12		
Lack of fit	0.19	3	0.062	0.36	0.7837
Pure error	0.68	4	0.17		
Cor total	27.89	16			
*R* ^2^	0.9690 0.9292 1.16 16.965				
*R* ^2^_adj_					
CV					
AP					

**Table 5:** The ANOVA results for the response surface quadratic models for the content of saponins extracted from the SPN.

Source (Content of Saponins)	Sum of Squares	DF	Mean Square	*F*-Value	*p*-Value
Model	406.03	9	45.11	47.91	<0.0001
*X*_1_	21.88	1	21.88	23.24	0.0019
*X*_2_	0.61	1	0.61	0.65	0.4472
*X*_3_	296.22	1	296.22	314.61	<0.0001
*X*_12_	14.67	1	14.67	15.58	0.0056
*X*_13_	0.055	1	0.055	0.059	0.8156
*X*_23_	8.97	1	8.97	9.53	0.0177
*X*_12_	22.69	1	22.69	24.10	0.0017
*X*_22_	1.64	1	1.64	1.74	0.2288
*X*_32_	37.24	1	37.24	39.55	0.0004
Residual	6.59	7	0.94		
Lack of fit	3.99	3	1.33	2.05	0.2493
Pure error	2.60	4	0.65		
Cor total	412.62	16			
*R* ^2^	0.9840 0.9635 1.43 24.331				
*R* ^2^_adj_					
CV					
AP					

**Table 6:** The ANOVA results for the response surface quadratic models for the antioxidant activity of saponins extracted from the SPN.

Source (Antioxidant Activity)	Sum of Squares	DF	Mean Square	*F*-Value	*p*-Value
Model	4.448 × 10^−3^	9	4.942 × 10^−4^	61.06	<0.0001
*X*_1_	2.611 × 10^−4^	1	2.611 × 10^−4^	32.25	0.0008
*X*_2_	2.556 × 10^−5^	1	2.556 × 10^−5^	3.16	0.1188
*X*_3_	3.089 × 10^−3^	1	3.089 × 10^−3^	381.62	<0.0001
*X*_12_	5.112 × 10^−5^	1	5.112 × 10^−5^	6.32	0.0402
*X*_13_	3.600 × 10^−5^	1	3.600 × 10^−5^	4.45	0.0729
*X*_23_	8.281 × 10^−5^	1	8.281 × 10^−5^	10.23	0.0151
*X*_12_	2.154 × 10^−4^	1	2.154 × 10^−4^	26.61	0.0013
*X*_22_	7.645 × 10^−6^	1	7.645 × 10^−6^	0.94	0.3635
*X*_32_	6.451 × 10^−4^	1	6.451 × 10^−4^	79.69	<0.0001
Residual	5.666 × 10^−5^	7	8.094 × 10^−6^		
Lack of fit	4.237 × 10^−5^	3	1.412 × 10^−5^	3.95	0.1087
Pure error	1.429 × 10^−5^	4	3.572 × 10^−6^		
Cor total	4.505 × 10^−3^	16			
*R* ^2^	0.9874 0.9713 5.67 27.165				
*R* ^2^_adj_					
CV					
AP					

## Abbreviations

ANOVA	analysis of variance
AP	adequate precision
APH	acetylphenylhydrazine
BBD-RSM	Box-Behnken design response surface methodology
CTX	cyclophosphamide
Hb	hemoglobin
PLT	platelet
PN	*Panax notoginseng*
PRESS	prediction error sum of squares
*R* ^2^	regression coefficient
RBC	red blood cell
SPN	steamed *Panax notoginseng*
WBC	white blood cess
